# The Registration of Nursing Homes

**Published:** 1907-11-23

**Authors:** 


### Nursing Administration.

### THE REGISTRATION OF NURSING HOMES.

### II. A QUESTION FOR THE MEDICAL PROFESSION.

The extent to which medical men are prejudiced in the exercise of their profession by bad nursing homes is hardly to be estimated. The physician or surgeon with a wide practice is constantly at his wits' end to provide places in reputable nursing homes for patients who cannot properly be nursed in their own houses; and since the homes which can absolutely be depended on are few in number, he is frequently compelled to fall back on those which are not so good. Now the nursing home which is "indifferent good" is to all practical intents and purposes very bad. The strength of a chain is its weakest link, and in choosing a place for the performance of an important operation, and for subsequent recovery, the existence of any weak link means serious jeopardy to the life of the patient. If this is not so, of what use the precautions which attend the treatment of disease in the public hospitals? Every one of those precautions, it is well known, have been proved to be necessary by bitter experience in the dangers of neglecting them. Then to what perils is the surgeon exposed when he is forced into the position of ignoring them all in his private practice? Nay, more, to what straits is the physician reduced when he is compelled to prescribe some form of treatment demanding exclusion of the patient from his ordinary surroundings, and yet is unable to place his patient in the position of getting it carried out with reasonable exactitude? It is these considerations which drive the medical man to invest capital in nursing homes, partly at any rate, controlled by himself. In theory it may seem an acceptable plan for a surgeon or physician to supervise a home in which his own patients can be received. Will not the arrangements in such a house leave nothing to be desired? It is probable that when the rare qualities of a good administrator are combined with the rare qualities of the good physician or surgeon, the result will be the best thing in private nursing homes to be had. But this combination of qualities is, indeed, rare. The claims of a man's profession, complicated as they often are by the linking of hospital with private practice, leave a very small margin of time which can be bestowed on the all-important work of supervision. If the matron be an exceedingly competent woman, who has herself had some experience in hospital administration, not merely in hospital nursing, all will be well. It is to such able superintendents, backed up by the advice of keen practitioners, that most of the successes in this line are due. But far more commonly when a doctor has invested money in a home it merely tells for harm. He is in a manner tied to send his patients there; he is disposed to think his own home nears perfection, and he turns a deaf ear to indications that all is not as it should be. Certainly, unless he is much unlike the average man, he will not receive with enthusiasm intimations that the whole system of drainage is antiquated, or that more capital is urgently required for theatre equipment. Some of the worst instances of mismanagement which are known to exist in this connection are due to this species of financial alliance between the doctor and the nursing home. The interests at stake are, indeed, far too great to be properly undertaken by private persons. We are confronted with the problem by whom, then, should they be undertaken? Few people have any idea of the large amount of capital necessary to equip even a small nursing home, and of the financial responsibilities involved in its efficient upkeep. Some idea may be gained by studying the sums of money necessary to be raised even to provide an open-air sanatorium. No one in their senses nowadays hires an old house in the centre of London with the design of receiving patients as a charity; nor if they did would the poor be eager to accept free of charge faulty accommodation which the rich must purchase at a high price. And yet, on the other hand, no one ever dreams of finding a good site for a nursing home, and erecting on it a building approximating in any way to a modern hospital. The thing has been done once. It was done on the initiative of Sir Henry Burdett, in the establishment of Fitzroy House, and this Pay Hospital remains an object lesson in reforms which await accomplishment. Would capital be easy to raise for such an institution on a scale worthy of the size and wealth of London? It is much to be doubted. Meantime, supposing that many nursing homes were closed, as they must be when legislation is obtained to protect their patients from obvious dangers, there are the pay wards in hospitals. St. Thomas's, Guy's, the Great Northern Central, and many of the special hospitals already admit paying patients, but it is a long time since any forward movement has been made to extend the work of voluntary hospitals in this direction. It is a matter the advisability of which rests solely on the willingness of the authorities to admit outside medical men to attend their own patients. As we pointed out a short time back, occasion seems at this moment to offer at St. Mary's for placing this much-needed accommodation at the service of the public, and at the same time securing large additional funds for the hospital as a charity. It can do nothing but good that the public, the public which desires to pay and can pay, should be admitted within the walls of the hospitals they assist to support. But whether the number of beds available for paying patients does or does not undergo augmentation, it is a matter of vital importance that members of the medical profession should inaugurate a complete reform of the way in which their private patients are nursed. The blind confidence reposed by the public in the specialist and the operator is with rare exceptions completely justified. This is more often due, we fear, when science has done its utmost, to the irrepressible inclination in man to recover under even adverse conditions, than to the skill with which the nursing treatment is carried out in the ordinary nursing home.

